# The Mount Vernon Hospital: Country Branch

**Published:** 1902-05-17

**Authors:** 


### The Institutional Workshop.

### THE MOUNT VERNON HOSPITAL: COUNTRY BRANCH.

### LAYING OF THE FOUNDATION-STONE

On Tuesday afternoon, the 13th inst., the foundation-stone of the new sanatorium, or convalescent branch, in connection with the Mount Vernon Hospital was laid at Northwood, in Middlesex, by H.R.H. Princess Christian. Two special trains conveyed a large number of persons from and to London, and the mile or so of road between Northwood Station and the scene of the ceremony was gay with pennons and banners, which mingled picturesquely with the young green of the foliage. Fortunately the rain kept off, but if the bright sunshine with which the afternoon began had but continued it would have added to the enjoyment of the visitors, for some of the ladies looked very cold in their spring dresses. The ceremony, however, was not a long one, and was over in little more than half an hour. The Princess, who was accompanied by H.R.H. Princess Victoria of Schleswig-Holstein, arrived with an escort of the Second (County of London) Imperial Yeomanry, and a Guard of Honour was mounted by the Cadet Corps of Harrow School (Ninth Middlesex Rifle Volunteers). Lady Agneta Montague and Colonel G. G. Gordon, C.V.O., C.B., were in attendance. Most of the committee and medical and surgical staff were on the platform, as well as the Mount Vernon nurses in indoor uniform.

Two beautiful bouquets having been presented by a little boy and girl, Mr. B. A. Lyon, chairman of the committee of management, welcomed the Royal visitors on behalf of the governors, and gave a brief account of the progress recently made at the Mount Vernon Hospital. He reminded the Princess of her two visits there—once to unveil the memorial to the late Mrs. Rundell Charles and again in 1893 to open the middle block of the building. During the five following years progress had been slow, but since 1898 it had been quite phenomenal. A grant of £1,000 had been made by the Prince of Wales's Fund on condition that fifteen beds were added: this was done and about the same time the open-air treatment was begun. Three open-air balconies had since been built, in which 40 patients slept in all weathers. These were now being taken down, and the open-air section was being built in a permanent form. When that was completed 150 patients would be accommodated, and Northwood would accommodate 100 more, making a total of 250. When completed, the hospital would be the second largest in the Metropolis. It was well known how greatly the Princess was in harmony with the objects of the charity, and it was a matter of rejoicing that Her Royal Highness was able to do so much to cheer and gladden the hearts of those actively engaged in the work.

The address over, a number of ladies and children presented purses; the choir sang "O God, our help in ages past," and prayers were read by the Rev. E. B. Backhouse, vicar of Northwood. The hymn "Now thank we all our God" followed the stone-laying, and the Bishop of Kensington pronounced a benediction on the building. The band of the Grenadier Guards accompanied the hymns and gave a selection of music at intervals.

The spot on which the function took place was the site of the main block. The wards are arranged in linear series, all facing south, with a spacious cross-ventilated corridor on the northern side, off which the lavatory blocks are placed, being connected with it by another cross-ventilated corridor.

The administrative block is placed between the two wings—one for men and the other for women—and contains doctors' and matron's rooms, dispensary, library, and recreation rooms, and the accommodation for servants is on the top floor.

The nurses' home, kitchens, doctor's house, and secretary's offices are all separately placed at some distance from the main patients' block, and connected with it by a wide corridor. The patients' dining hall, which is common to both sexes, is also detached.

Pathological rooms, mortuary, laundries, and an electric lighting station will also be provided. Great consideration has been paid to the general planning and design of the buildings by the committee and architect, who have endeavoured to erect a sanatorium that shall fully meet all the modern requirements of the open-air treatment of consumption. The plans of Mr. Frederick Wheeler, F.R.I.B.A., are being carried out by Messrs. Holland and Hannen. The design for the patients' block is in the Royal Academy Exhibition. The site chosen, a prettily wooded park of 60 acres, is well away from the village, and commands beautiful views in whichever direction one looks.

It will be remembered that the cost of this new development is being defrayed by the donor of £121,000, who wishes to remain anonymous.

